# Deer antler reserve mesenchyme cells modified with miR-145 promote chondrogenesis in cartilage regeneration

**DOI:** 10.3389/fvets.2024.1500969

**Published:** 2024-12-24

**Authors:** Boyin Jia, Xintong Han, Xin Li, Linlin Zhang, Fuquan Ma, Yusu Wang, Xue Wang, Yaru Yan, Yaxin Li, Junnan Shen, Xinran Chen, Xinyi Li, Qianzhen Zhang, Pengfei Hu, Rui Du

**Affiliations:** ^1^College of Animal Medicine/College of Animal Science and Technology, Jilin Agricultural University, Changchun, China; ^2^Laboratory of Production and Product Application of Sika Deer of Jilin Province, Jilin Agricultural University, Changchun, China; ^3^Institute of Antler Science and Product Technology, Changchun Sci-Tech University, Changchun, China; ^4^Yanbian University, Yanbian, China

**Keywords:** reserve mesenchyme cells, SOX9, miR-145, chondrogenesis, cartilage regeneration

## Abstract

Deer antler-derived reserve mesenchyme cells (RMCs) are a promising source of cells for cartilage regeneration therapy due to their chondrogenic differentiation potential. However, the regulatory mechanism has not yet been elucidated. In this study, we analyzed the role of microRNAs (miRNAs) in regulating the differentiation of RMCs and in the post-transcriptional regulation of chondrogenesis and hypertrophic differentiation at the molecular and histological levels. The results showed that RMCs showed typical MSC differentiation potentials. During chondrogenic differentiation, we obtained the expression profile of miRNAs, among which miR- 145 was the most prominent candidate as a key microRNA involved in the balance of chondral and endochondral differentiation. Knockdown of miR-145 promoted chondrogenesis and inhibited hypertrophy differentiation in RMCs. Mechanically, by prediction through online databases combined with dual-luciferase reporter assay, SOX9 was suggested as a target of miR-145. Further validation experiments confirmed that knockdown of miR-145 contributed to the balance between endochondral versus chondral differentiation of RMCs by targeting SOX9. Additionally, RMCs transfected with the miR-145-knockdown-mediated lentiviral vector successfully promoted cartilage regeneration *in vivo*. In summary, our study suggested that the reciprocal negative feedback between SOX9 and miR-145 was essential for balancing between endochondral versus chondral differentiation of RMCs. Our study suggested that modification of RMCs using miRNAs transduction might be an effective treatment for cartilage defects.

## Introduction

Cartilage defect is a common orthopedic disease caused by trauma, necrosis, inflammation, and degeneration, which is an important cause of osteoarthritis (OA) (1). The lack of blood vessels in cartilage tissue and the inability of chondrocytes in the cartilage lacunae to migrate to the wound site make it difficult for cartilage to self-repair (2, 3). Mesenchymal stem cell (MSC) implantation is an important method for treating cartilage defects in tissue engineering. MSCs not only have the potential to differentiate into chondrocytes but also possess paracrine function, anti-inflammatory activity, immunomodulatory capacity (4). However, the phenotype of MSC-generated chondrocytes is unstable and undergoes hypertrophic degeneration, ultimately resulting in the formation of endochondral bone (5). In addition, the lifespan and proliferation potential of MSCs are limited, and they even have a tendency for tumor formation (6). Therefore, it is necessary to explore more sources of MSCs.

Antler is the fastest animal-growing tissue in mammals (2 cm/day) (7). The growth center of antler is located at its top, and the MSCs present in it are called reserve mesenchymal cells (RMCs) (8). RMCs can differentiate into chondrocytes, and despite high cell division rate, they do not undergo cancerous transformation (9). Accumulating evidence demonstrates that antler has evolved efficient cell apoptosis in the reserve mesenchymal, offsetting the tendency toward neoplastic transformation (9). It can be seen that RMCs effectively avoid the neoplastic tendencies of previous MSCs, thereby reducing the risk of treating cartilage defects in clinical practice. In our previous study, we have successfully repaired cartilage defects in rat models using RMCs (10). Therefore, RMCs will become a potential cellular resource for the treatment of cartilage defects.

Increasing evidence suggests that the post-transcriptional regulation of miRNAs can target transcription factors to determine cell fate (11). miRNAs play a crucial role in cell differentiation, development, proliferation, and other processes (12). miRNAs also regulate the differentiation of MSCs into chondrocytes, such as miR-140 targets RALA to promote chondrogenesis of bone marrow-derived MSCs (BMSCs), while targeting BMP2 inhibits the osteogenic effect of BMSCs (13, 14). miR-140-transfected umbilical cord MSCs (UC-MSCs) effectively repair cartilage repair in an OA rat model (15). miR-199b-5p targets JAG1 and stimulates chondrogenesis of BMSCs by increasing SOX9 and COL2 protein levels (16). miR-181a targets RSPO2 to activate BMP signaling pathway and reduce typical WNT signaling pathway in BMSCs (17). In our previous study, we have identified a group of differentially expressed miRNAs in antler growth centers at four different developmental stages but did not characterize the target mRNAs (18). We have noticed the differentially expressed miR-145 among them. The study has found that miR-145 can directly target SOX9 in the initial stage of chondrogenic differentiation of MSCs, thereby inhibiting cartilage formation. On the contrary, knockdown of miR-145 can increase the expression of SOX9, thereby delaying the differentiation process of MSCs into hypertrophic chondrocytes (19). SOX9, as an essential gene for chondrogenesis, transcriptionally activates the genes for many cartilage-specific structural component (20). Unfortunately, it is still unclear which miRNAs are involved in post-transcriptional regulation of the chondrogenic differentiation of RMCs, especially the important role of miR-145 in this process.

In this study, we screened miRNAs directly involved in post-transcriptional regulation of RMC chondrogenic differentiation-related genes by miRNA expression profiles. We identified miR-145 as the optimal candidate for targeting chondrogenic genes and further determined the expression pattern and function of miR-145 during RMCs chondrogenic differentiation. This will help improve the efficacy of RMC-derived chondrocytes in repairing cartilage defects.

## Materials and methods

### Tissue collection and cell cultures

All animal experiments were performed as per the laboratory animal—guidelines for ethical review of animal welfare (GBT 35892.-2018) and were approved by the Jilin Agricultural University Committee on the use of live animals (Approval No: 20220311008). The sika deer was procured from Sika Deer Breeding Farm of Jilin Agricultural University. Healthy adult male sika deer (3 years old; *n* = 6) was used for this study. The sika deer was anesthetized by intramuscular injection of 50 mg/kg chlorpromazine hydrochloride. The sika deer was awakened at the end of experiments with 0.075 mL/kg composite awakening agent consisting of atemizole, naloxone, and flumazenib (0.90:0.95:0.26). Antler tips were collected from six anesthetized male 3-year-old sika deer. According to the previously reported method, the tissues of reserve mesenchyme and cartilage were collected from the growing antler tips at 30 days and 60 days (21). The culture of RMCs and antler chondrocytes (CC) was determined following Li et al. (22). Three biological replicate experiments for each tissue type were performed.

### Flow cytometry

Flow cytometry was used to identify the surface markers of RMCs as previously described (10). In brief, the RMCs were mixed with different fluorescently labeled monoclonal antibodies, including CD34, CD45, CD73, and CD90. After washing the RMCs, we immediately determined them using a flow cytometer.

### Immunofluorescent staining

The RMCs on 24-well plates were fixed with 4% paraformaldehyde and permeabilized by 0.5% Triton X-100. After being blocked by serum, the cells were incubated overnight with primary antibodies (CD34, CD45, CD73, CD90). Then, the RMCs were incubated for 1 h with the secondary antibodies. Finally, the nucleus was stained with DAPI, and the images were analyzed using a fluorescence microscope.

### Multilineage differentiation

We used a chondrogenic differentiation kit to determine the chondrogenic ability of RMCs. In short, 500,000 cells were placed in centrifuge tubes. Then the culture medium was changed from maintenance medium to chondrogenic medium, changing every 2 days for a course of 14 days. The final formed cartilage-like nodules were embedded in optimal cutting temperature (OCT), cut into 5-μm frozen sections, and stained with Alcian blue. MesenCult adipogenic differentiation kit and osteogenic differentiation kit were used to determine the adipogenic and osteogenic abilities of RMCs. A 12-well plate was cultured with 200,000 RMCs/well. Then the culture medium was changed from maintenance medium to adipogenic medium or osteogenic medium, changing every 3 days. After 14 days, oil red O was used to stain lipid droplets of adipocytes. After 21 days, Alizarin Red was used to stain calcium deposits of osteoblasts.

### Quantitative real-time PCR (qRT-PCR)

Total RNA from cells and cartilage-like nodules was isolated using RNA-quick purification kit. Isolated RNAs were reverse-transcribed into cDNAs with the use of PrimeScript^™^ RT reagent kit, and cDNA was amplified with the use of TB Green^®^Premix Ex Taq^™^ II. The primer sequences are shown in Supplementary File S1. Relative expression levels of miRNAs and mRNAs were normalized to U6 and GAPDH internal control.

### Western blot

RIPA lysate (Beibokit, Shanghai, China) was utilized for extracting total proteins from cells and cartilage-like nodules. Quantification of proteins was achieved using a BCA protein kit assay. Protein was separated by 10% sodium dodecyl sulfate-polyacrylamide gel electrophoresis (SDS-PAGE) and transferred to polyvinylidene difluoride (PVDF) membranes, which were subsequently blocked with 5% skimmed milk. The PVDF membranes were incubated with primary antibodies on a shaker overnight at 4°C. The membranes were subsequently incubated with secondary antibodies at ambient temperature. The protein was detected using ECL detection reagents (Tanon, Shanghai, China) and signal was normalized to β-actin.

### MiRNA sequencing

Total RNA was collected from chondrocytes and RMC-induced chondrogenic differentiation was done for 0, 7, and 14 days to construct small RNA libraries. Small RNAomics were sequenced on the Illumina Novaseq 6000. The small RNA libraries were constructed and sequenced according to the method described previously (18). The differential expression miRNAs (DE miRNAs) were obtained with *p* < 0.05 and fold-change cutoff of two. Online databases including miRmap, TargetScan, and miRanda were used to predict the targets of miRNAs. A Venn diagram was constructed to display overlapping interactions.

### Transfection procedure

miR-145-overexpression lentivirus, miR-145-knockdown lentivirus, scrambled miRNA controls lentivirus, SOX9-overexpression lentivirus, and SOX9-knockdown lentivirus were purchased from Hanbio Biotechnology Co. Ltd. (Shanghai). The transfection process of lentivirus followed the manufacturer’s instructions. About 2 × 10^5^ RMCs were inoculated overnight in a 12-well plate and infected with lentivirus expressing either target gene lentivirus at a multiplicity of 50 after 48 h for further testing.

### Dual-luciferase reporter assay

The binding sequences of miR-145 and SOX9 3’UTR were predicted according to targetscan database. The SOX9-3’untranslated region-wild type and SOX9-3’untranslated region-mutant type in luciferase reporter plasmids were constructed. Then, the luciferase activity was measured after co-transfection with the plasmids and miR-145 mimic or miRNA-NC in 293 T cells.

### Animal experiments

Sprague Dawley (SD) rats were procured from Experimental Animal Center of Jilin Agricultural University. The animals were kept in a standard laboratory condition. Healthy adult male rats (weight: 250 ± 30; 8 weeks old; *n* = 48) were used for this study. The rats were anesthetized by intraperitoneal injection of 20% urethan (1 g/kg). The rats were euthanized at the end of experiments with 20% urethan (4 g/kg). The rats were randomly divided into miR-145 mimic group, miR-145 inhibitor group, miR-145 NC group, RMCs group, PBS group, and Sham group to establish a cartilage defect model. The rat articular cartilage defect model was produced by our previous description (10). miR-145 mimic group received RMCs transfected with miR-145-overexpression lentivirus (10^6^ cells/joint). miR-145 inhibitor group received RMCs transfected with miR-145-knockdown lentivirus (10^6^ cells/joint). miR-145 NC group received RMCs transfected with miR-145-NC lentivirus (10^6^ cells/joint). The RMC group received RMCs (10^6^ cells/joint). The PBS group received 100 μL PBS. Sham control animals only underwent surgery without treatment once a week for 3 weeks. The grip strength level of rats was investigated using the grip strength test. After 8 weeks of treatment, the maximum pulling force of the hind limbs was measured five consecutive times, and the average value was recorded. At 4 and 8 weeks after surgery, the femurs of rats were collected for the evaluation of disease progression. The macroscopic evaluation of cartilage defects was made according to the Innovative Clinical Research Solutions (ICRS) criteria.

### Histological examination

Hematoxylin and eosin (HE) staining (Bioss, Beijing, China), Safranin-O-Fast green staining (Phygene, Fuzhou, China), and IHC reagent kit (ZSGB Bio, Beijing, China) were used to assess the histological changes in sampled femurs. Briefly, the whole femurs were incubated in 4% paraformaldehyde for 1 day and decalcified for 1 month using 10% EDTA. All samples were embedded in paraffin blocks and sectioned at a thickness of 4 μm. Then, sections were stained with HE and Safranin-O-Fast green. The methods of Pineda and Wakitani were used to score the severity of the degree of cartilage defects. The parameters of Pineda score included percent filling of the defect, reconstitution of the osteochondral junction, matrix staining, and cell morphology. The range was from the best 0 to the worst 14 (23). The parameters of Wakitani score included cell morphology, matrix-staining, surface regularity, thickness of cartilage, and the integration of donor with host. The range was from the best 0 to the worst 14 (24). For immunohistochemical analysis, the tissue sections were incubated with primary antibodies overnight at 4°C. Horseradish peroxidase-labeled secondary antibody was incubated at 37°C for 60 min. The color was developed by applying 3,3′-diaminobenzidine. The sections were counterstained with hematoxylin.

### Statistical analysis

All statistical analyses were conducted with SPSS 26. The means of two groups were compared using two-tailed independent Student’s *t*-test, and the means of multiple groups were compared by one-way analysis of variance (ANOVA). The difference was considered statistically significant at *p* value < 0.05 (*p < 0.05, **p < 0.01, ***p < 0.001).

## Results

### Characteristics of RMCs and chondrocytes

**Figure 1:**
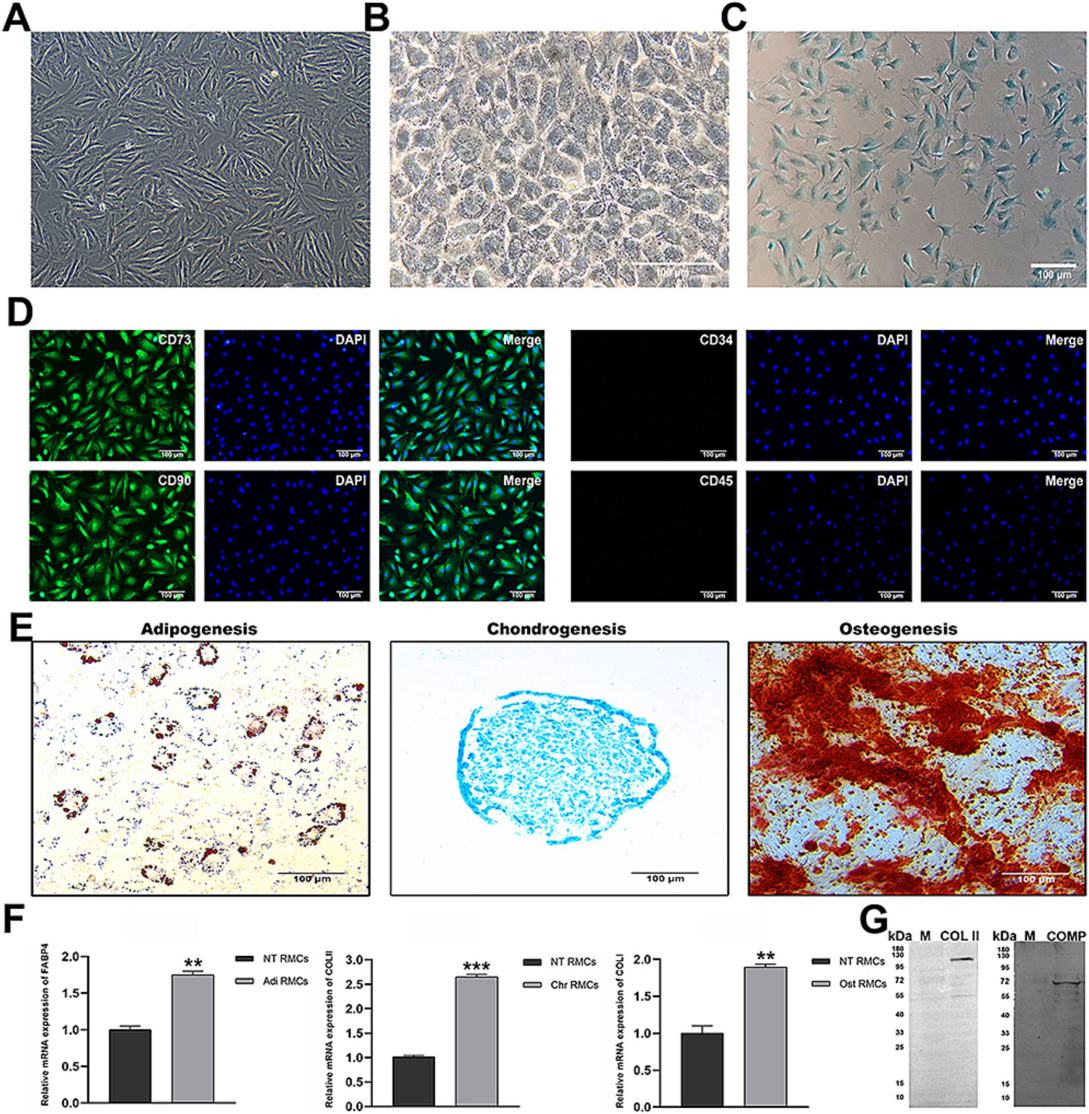
Identification of characteristics of RMCs and chondrocytes. **(A)** Morphology of RMCs. **(B)** Morphology of chondrocytes. **(C)** Alisin blue staining of chondrocytes. **(D)** IF analysis of RMCs surface markers. **(E)** Multilineage differentiation capacity. **(F)** Analysis of adipogenic, chondrogenic, and osteogenic gene expression after multilineage differentiation of RMCs by qRT-PCR. **(G)** Analysis of chondrogenic protein expression by WB.

Firstly, we evaluated whether the cultured RMCs exhibited the characteristics of MSCs. RMCs had spindle-shaped, fibroblast-like morphology (Figure 1A). The surface marker profile of RMCs was confirmed by immunofluorescent staining, including the expression of CD73 and CD29 (Figure 1D). Following the induction of adipogenic, chondrogenic, or osteogenic differentiation, the RMCs were positively stained for oil red O, Alcian blue, or Alizarin red, respectively (Figure 1E). Furthermore, real-time quantitative reverse transcription polymerase chain reaction (qRT-PCR) showed an increased expression of adipogenic, chondrogenic, and osteogenic genes of RMCs after designated induction (Figure 1F). These results showed that RMCs exhibited typical characteristics of MSCs. Secondly, we evaluated the basal characteristics of antler chondrocytes. They resembled paving stones in morphology (Figure 1B). Alisin blue staining of antler chondrocytes was positive (Figure 1C), and the expression of chondrogenic markers COL II and COMP was further detected by WB (Figure 1G). These results indicate that the cultured cells in this study exhibited typical chondrocyte characteristics.

### miR-145 was differentially expressed during the chondrogenic differentiation of RMCs

**Figure 2:**
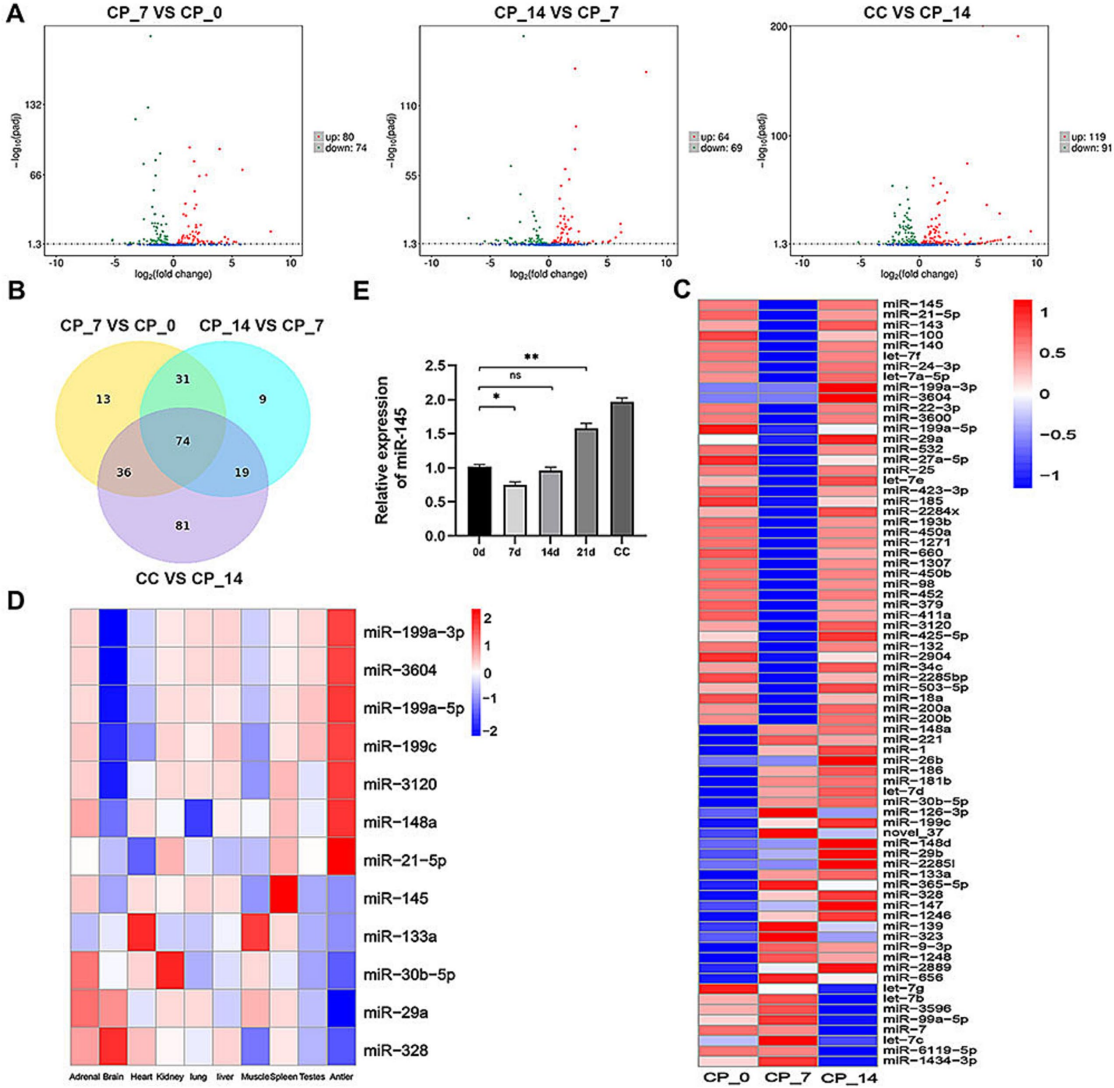
miR-145 was assumed as a putative miRNA that might participate in the chondrogenic differentiation of RMCs. **(A)** Volcano map of differentially expressed miRNAs in the pairwise comparisons. **(B)** Venn diagram of differentially expressed miRNAs in the pairwise comparisons. **(C)** Clustering heatmap of 74 differentially co-expressed miRNAs. **(D)** Clustering heatmap of 12 candidate miRNAs related to antler growth. **(E)** Analysis of miR-145 expression after chondrogenic differentiation of RMCs by qRT-PCR. CP_0: RMCs induced chondrogenic differentiation for 0 days; CP_7: RMCs induced chondrogenic differentiation for 7 days; CP_14: RMCs induced chondrogenic differentiation for 14 days; CC: chondrocytes.

miRNA sequencing was used to identify and select the differentially expressed miRNAs from chondrocytes and RMC-induced chondrogenic differentiation for 0, 7, and 14 days. A total of 475 known miRNAs were identified, of which 240 were differentially expressed (Figure 2A). The expression levels of 74 differentially co-expressed miRNAs in all comparison groups were more than two-fold different (Figure 2B). Hierarchical clustering analysis was performed to display the dynamic profiles of 74 differentially expressed miRNAs in Figure 2C. Based on our previous miRNA sequencing results on 10 different tissues of sika deer, we identified 12 candidate miRNAs related to antler growth (Figure 2D). Among them, miR-145 was well-known for regulating the proliferation and differentiation of chondrocytes. We further identified the dynamic expression of miR-145 by qRT-PCR (Figure 2E). This expression trend was consistent with miRNA sequencing. Therefore, we speculated that miR-145 might play a crucial role in the chondrogenic differentiation of RMCs.

### Low expression of miR-145 promoted chondrocyte differentiation of RMCs

**Figure 3:**
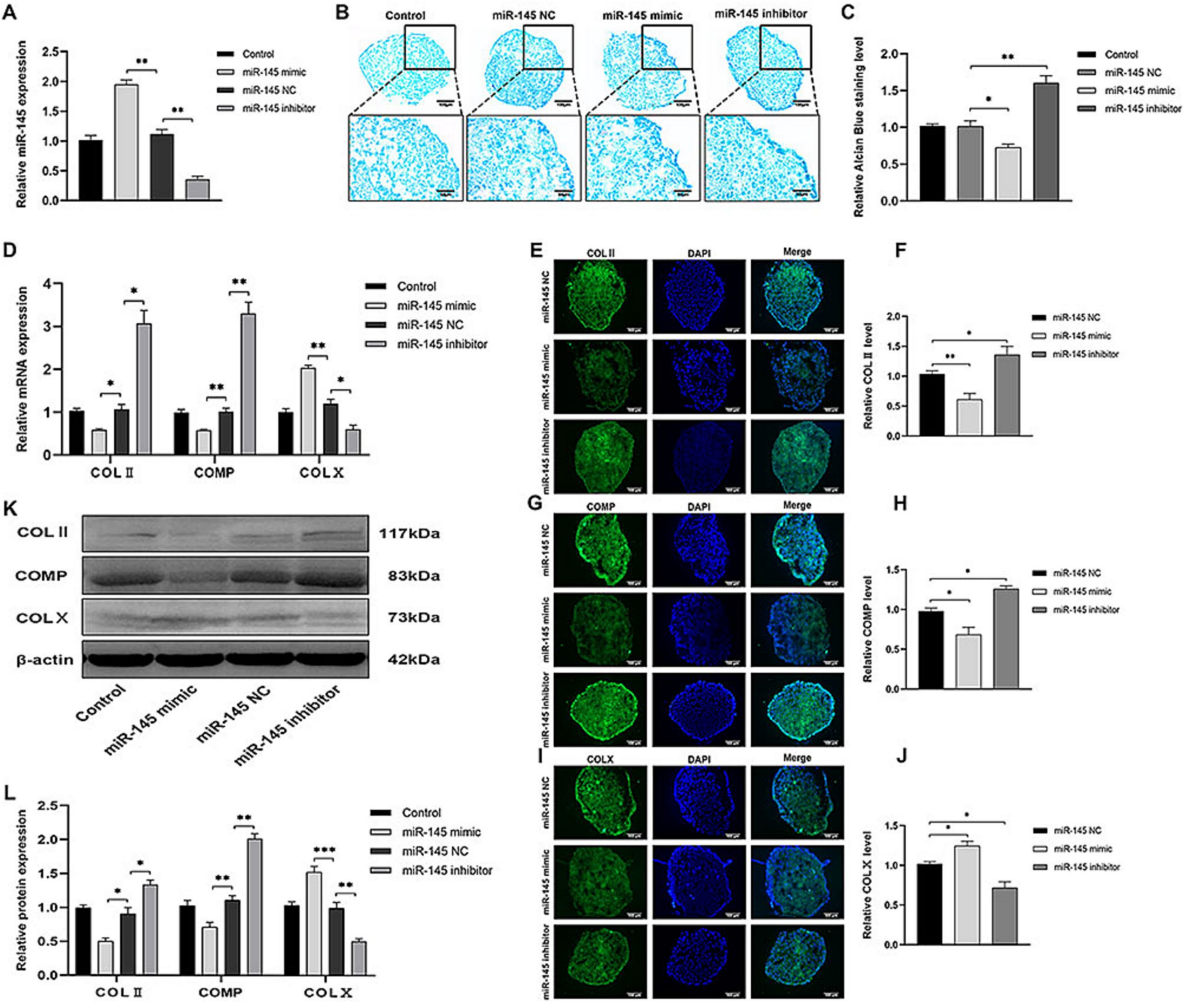
miR-145 inhibited chondrocyte differentiation of RMCs. **(A)** The relative expression of miR-145 in RMCs with different treatments was evaluated by qRT-PCR. **(B)** Alisin blue staining of RMCs with different treatments. **(C)** Quantitative analysis of Alcian blue staining. **(D)** The expression of chondrogenic genes of RMCs with different treatments was analyzed using qRT-PCR. **(E)** IF of COL II of the RMCs with different treatments. **(F)** Quantitative analysis of IF of COL II. **(G)** IF of COMP of the RMCs with different treatments. **(H)** Quantitative analysis of IF of COMP. **(I)** IF of COL X of the RMCs with different treatments. **(J)** Quantitative analysis of IF of COL X. **(K)** The expression of chondrogenic proteins of RMCs with different treatments was analyzed using WB; **(L)** Quantitative analysis of WB.

Lentivirus was selected for RMC transfection to increase or reduce miR-145 expression. Chondrogenic differentiation was induced in RMCs with different treatments by pellet culture for 14 days. It was confirmed that miR-145 mimic group showed a significant increase in miR-145 expression, while miR-145 inhibitor group showed a significant decrease in miR-145 expression by qRT-PCR (Figure 3A). Histological analysis of pellets displayed that the staining of glycosaminoglycan in miR-145 inhibitor group using Alcian blue was stronger than control group, while the results of the miR-145 mimic group were opposite (Figures 3B,C). The expression of chondrogenic and hypertrophic markers in RMC after different treatments was detected through qRT-PCR, IF, and WB. The results consistently showed that the expression of COL II and COMP decreased in the miR-145 mimic group, while the expression of COL X increased. On the contrary, the expression of COL II and COMP increased in the miR-145 inhibitor group, while the expression of COL X decreased (Figures 3D–L). Thus, it can be concluded that knockdown of miR-145 may effectively promote the chondrocyte differentiation of RMCs, while overexpression of miR-145 may promote hypertrophic differentiation after chondrogenic induction in culture.

### SOX9 was a target gene of miR-145

**Figure 4:**
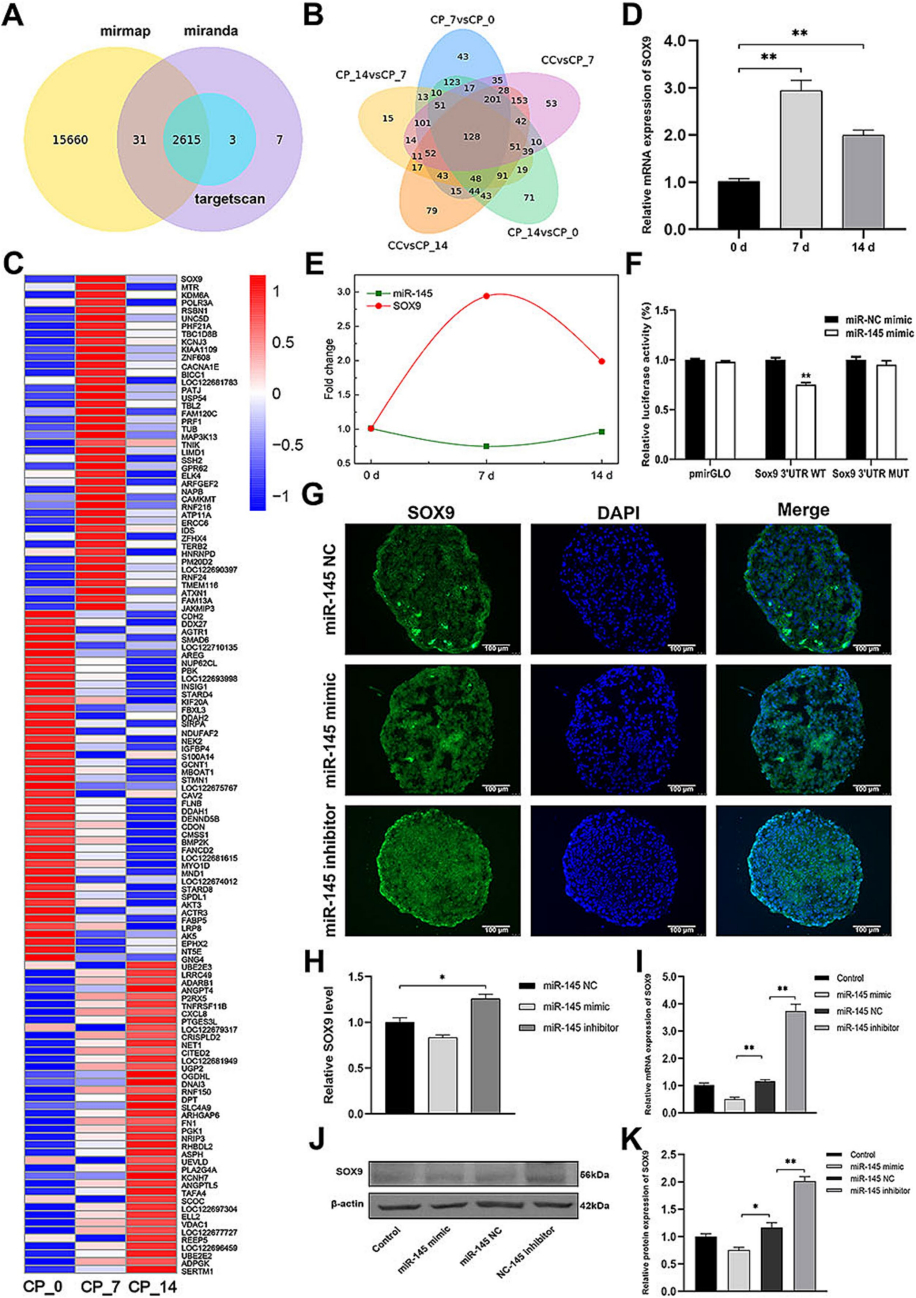
miR-145 targeted and suppressed SOX9 expression. **(A)** The target genes of miR-145 were predicted by online websites. **(B)** Venn diagram of differentially expressed target genes of miR-145 in the pairwise comparisons. **(C)** Clustering heatmap of 128 differentially co-expressed target genes of miR-145. **(D)** qRT-PCR analysis of SOX9 expression during RMC chondrogenic differentiation. **(E)** The expression trend of miR-145 and SOX9 was negatively correlated. **(F)** Normalized luciferase activity after co-transfection of miR-NC mimics or miR-145 mimics together with SOX9 3’UTR-WT or SOX9 3’UTR-MUT. **(G)** The expression of SOX9 of RMCs with different treatments was analyzed using IF. **(H)** Quantitative analysis of IF. **(I)** The expression of SOX9 of RMCs with different treatments was analyzed using qRT-PCR. **(J)** The expression of SOX9 of RMCs with different treatments was analyzed using WB. **(K)** Quantitative analysis of WB.

Using miRmap, TargetScan, and miRanda databases to identify putative target genes of miR-145, we obtained 2,615 intersecting target genes (Figure 4A). Then, the intersected target genes were compared with the differentially expressed genes in the CP_7 vs. CP_0, CP_14 vs. CP_7, CP_14 vs. CP_0, CC vs. CP_7, and CC vs. CP_14 groups, and 128 intersected candidate target genes were further identified (Figure 4B). Among them, 43 intersecting candidate target genes showed a significant negative correlation with the expression of miR-145 during chondrogenic differentiation of RMCs (Figure 4C). According to previous reports, SOX9 and KDM6A were closely related to chondrogenesis, and SOX9 was particularly important as a switch for MSCs to induce chondrogenic differentiation (25, 26). Therefore, we further validated the expression of SOX9 during chondrogenic differentiation of RMCs, and qRT-PCR results showed that the expression trend of SOX9 was opposite to that of miR-145 (Figures 4D,E). Moreover, using dual-luciferase reporting system, we found that miR-145 mimic could significantly reduce the luciferase activity of the tested SOX9 3′UTR reporter (Figure 4F). These data demonstrated that miR-145 can directly target 3′UTR of SOX9. The empty vector or recombinant lentivirus containing the entire coding sequence of miR-145 or miR-145 inhibitor was transfected into RMCs. Then, the SOX9 expression was measured after inducing chondrogenic differentiation of RMCs for 14 days using qRT-PCR, IFA, and WB. The results consistently showed that overexpression of miR-145 inhibited the expression of SOX9, while knockdown of miR-145 promoted the expression of SOX9 (Figures 4G–K). Therefore, it was speculated that miR-145 regulates the chondrogenic differentiation process in RMCs by targeting SOX9.

### miR-145-targeted SOX9 to inhibit chondrogenic differentiation of RMCs

**Figure 5:**
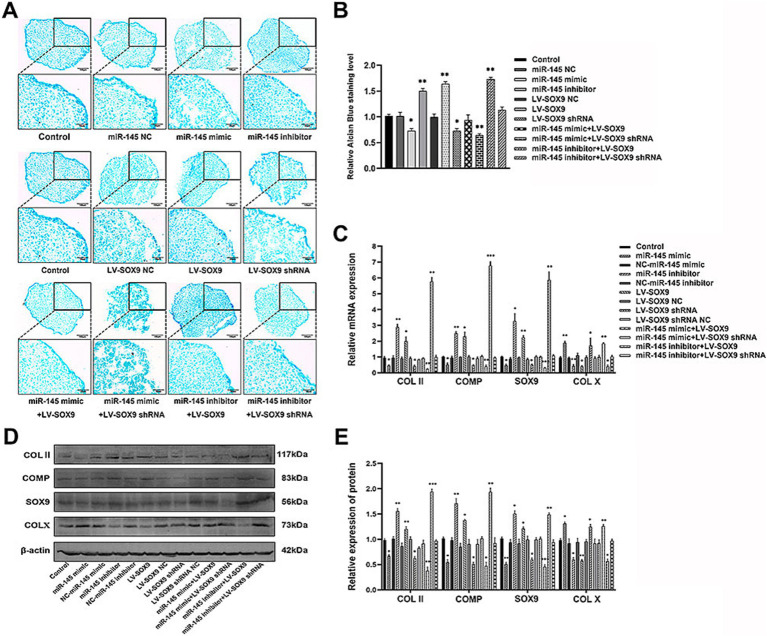
MiR-145-targeted SOX9 to inhibit chondrogenic differentiation of RMCs. **(A)** Alisin blue staining of RMCs with different treatments. **(B)** Quantitative analysis of Alcian blue staining. **(C)** The expression of chondrogenic genes of RMCs with different treatments was analyzed using qRT-PCR. **(D)** The expression of chondrogenic proteins of RMCs with different treatments were analyzed using WB. **(E)** Quantitative analysis of WB.

**Figure 6:**
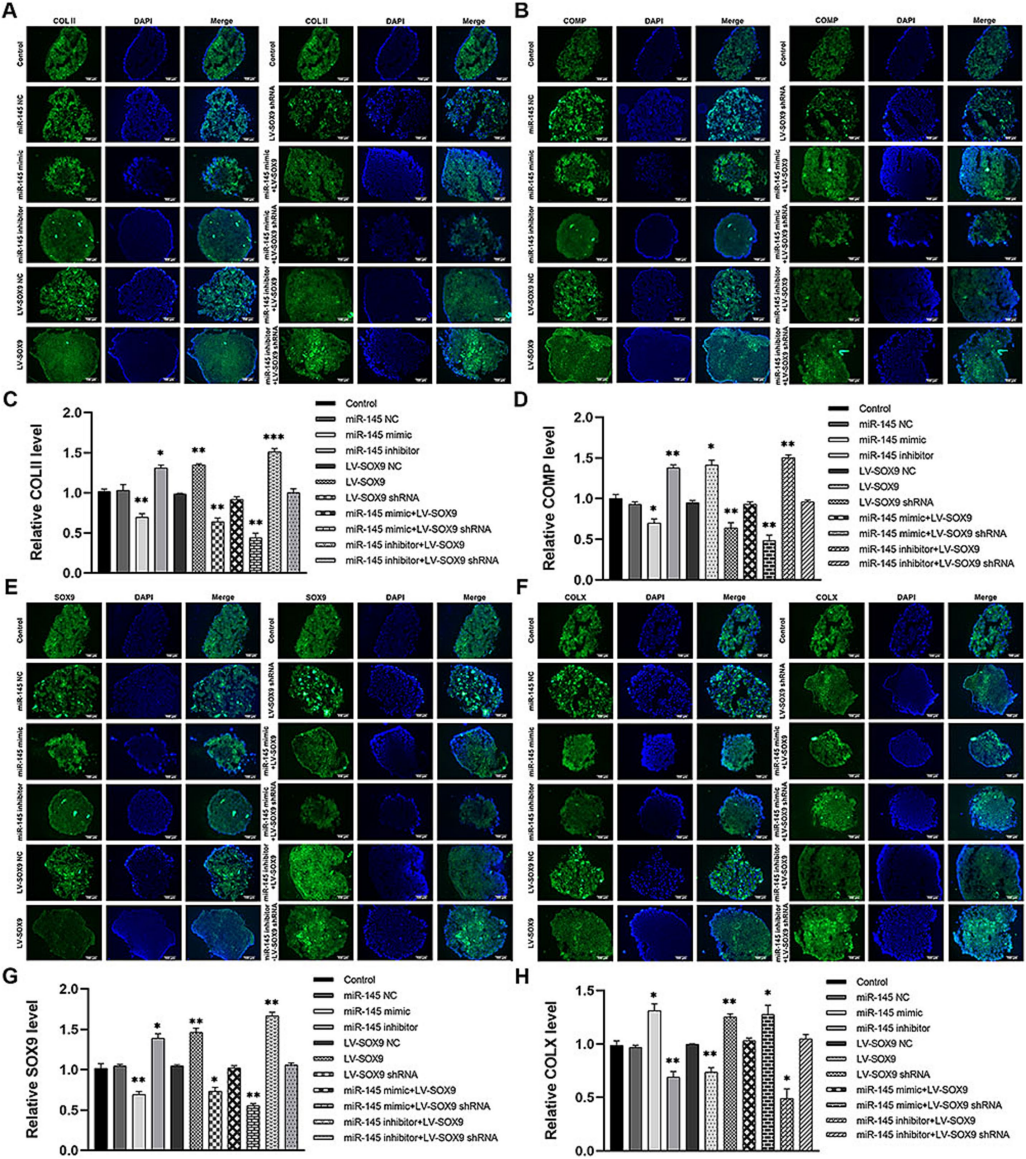
The expression of chondrogenic and hypertrophic markers in RMCs transfected with different lentiviruses were identified using IF. **(A)** IF of COL II of the RMCs with different treatments. **(B)** Quantitative analysis of IF of COL II. **(C)** IF of COMP of the RMCs with different treatments. **(D)** Quantitative analysis of IF of COMP. **(E)** IF of SOX9 of the RMCs with different treatments. **(F)** Quantitative analysis of IF of SOX9. **(G)** IF of COL X of the RMCs with different treatments. **(H)** Quantitative analysis of IF of COL X.

To verify whether miR-145 regulated the chondrogenic differentiation of RMCs through SOX9, the empty vector or recombinant lentivirus containing the entire coding sequence of miR-145, miR-145 inhibitor, SOX9, and SOX9 shRNA was transfected into RMCs. The results of Alcian blue staining demonstrated the following: miR-145 knockdown or SOX9 overexpressing increased the staining intensity; miR-145 overexpressing or SOX9 knockdown decreased the staining intensity; SOX9 knockdown reversed the promotion effect of the low expression of miR-145 on glycosaminoglycan deposition; SOX9 overexpressing rescued the inhibitory effect of miR-145 overexpression on glycosaminoglycan deposition (Figures 5A,B). Furthermore, the results of qRT-PCR, IF, and WB highlighted that the overexpression of miR-145 knockdown or SOX9 promoted the expression of COL II, COMP, and SOX9, while such overexpression inhibited the expression of COL X. On the contrary, miR-145 overexpression or SOX9 knockdown inhibited the expression of COL II, COMP, and SOX9 but promoted the expression of COL X. SOX9 knockdown reversed the promoting effect of miR-145 low expression on the chondrogenesis of RMCs, and SOX9 overexpression rescued the inhibitory effect of miR-145 overexpression on the chondrogenesis of RMCs (Figures 5C–E, 6A–H). Overall, miR-145 targeted SOX9 to inhibit the chondrogenic differentiation process in RMCs.

### Low expression of miR-145 in RMCs enhanced the repair efficacy of cartilage injury

**Figure 7:**
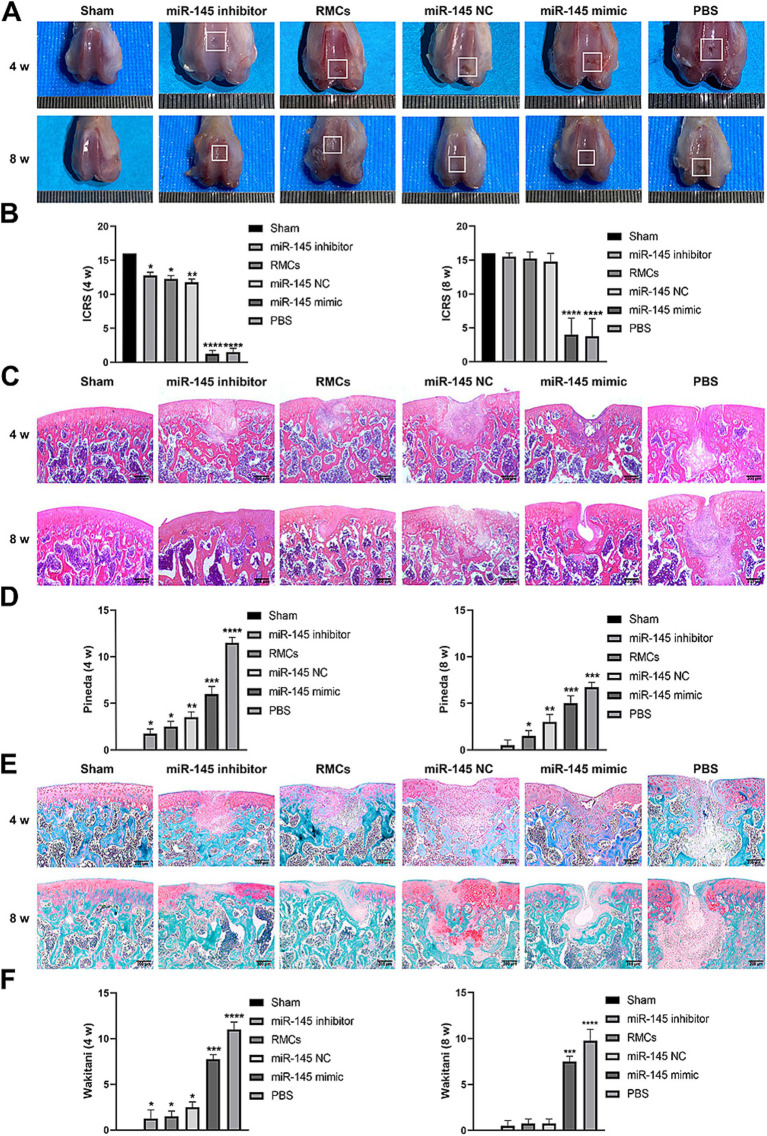
Low expression of miR-145 enhanced the repair efficacy of the RMCs and reduced their hypertrophic differentiation in the rat cartilage injury model. **(A)** Femoral was evaluated by Gross appearance. **(B)** Macroscopic ICRS scores of the femur. **(C)** Femoral section was evaluated using HE staining. **(D)** Pineda scoring of the HE staining. **(E)** Femoral section was evaluated using Safranin-O-Fast green staining. **(F)** Wakitani scoring of the Safranin-O-Fast green staining.

**Figure 8:**
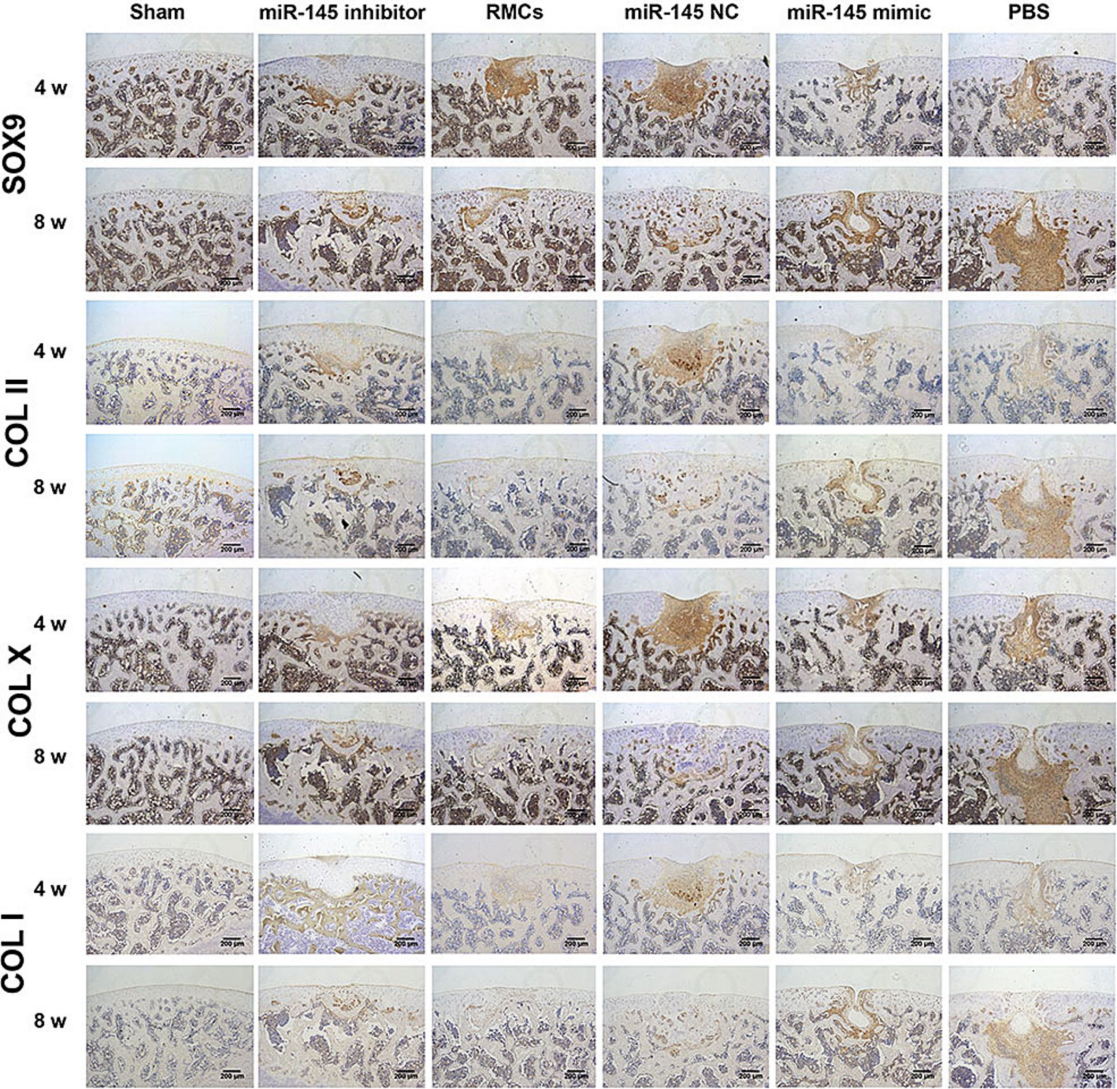
Immunohistochemical staining of SOX9, COL II, COL X, and COL I in femoral sections to evaluate repair efficacy.

The role of miR-145 in regulating cartilage repair by RMCs on rats with full-thickness cartilage defect was evaluated. The *in vivo* models were injected with RMCs treated with miR-145 mimic, miR-145 inhibitor, and miR-NC. At the macroscopic level, 8 weeks after operation, grip strength testing shown that RMC treatment could significantly improve the hind limb grip level of rats (Supplementary Figure S1). The defect of miR-145 inhibitor group was filled with newly formed tissue, appearing smooth on the surface. The defects of RMCs group and miR-NC group were also filled with the newly formed tissue but with clear boundaries from the surrounding tissue. The defects of miR-145 mimic group and PBS group were only partially filled (Figure 7A). The ICRS scores showed that the miR-145 inhibitor group had the highest score, followed by the RMCs group and miR-NC group, while the miR-145 mimic group and PBS group had the lowest scores (Figure 7B). At the histological level, HE staining and Safranin-O-Fast green staining results revealed that the top layer cartilage of the miR-145 inhibitor group was perfectly integrated with the adjacent cartilage, and well-differentiated chondrocytes were surrounded by abundant matrix proteoglycan. In contrast, the cartilage in the miR-145 mimic group exhibited poor integration with the adjacent cartilage, characterized by a reduction in chondrocyte clusters and a loss of matrix proteoglycans (Figures 7C,E). According to the histological assessment by Pineda and Wakitani, the miR-145 inhibitor group had the lowest score, followed by the RMC group and miR-NC group, while the miR-145 mimic group and PBS group had the highest scores (Figures 7D,F). At the molecular level, the qRT-PCR results confirmed a significant change in the miR-145 expression after treatment with miR-145-modified RMCs (Supplementary Figure S2). The immunohistochemical results demonstrated that the expression of fibrous chondrocyte markers COL I and COL X was strong in the miR-145 mimic group, while the expression of SOX9 and COL II was lower (Figure 8). On the contrary, the expression of COL I and COL X was downregulated in the miR-NC and miR-145 inhibitor groups, while the expression of SOX9 and COL II was upregulated (Figure 8). This indicated that miR-145 tended to promote hypertrophy differentiation and endochondral bone formation of RMC-derived chondrocytes *in vivo*. In summary, the histological staining results were consistent with the protein expression of SOX9, COL II, COL X, and COL I. These data suggested that a low expression of miR-145 effectively enhanced the repair efficacy of the RMCs and reduced their hypertrophic differentiation in the cartilage defect model.

## Discussion

**Figure 9:**
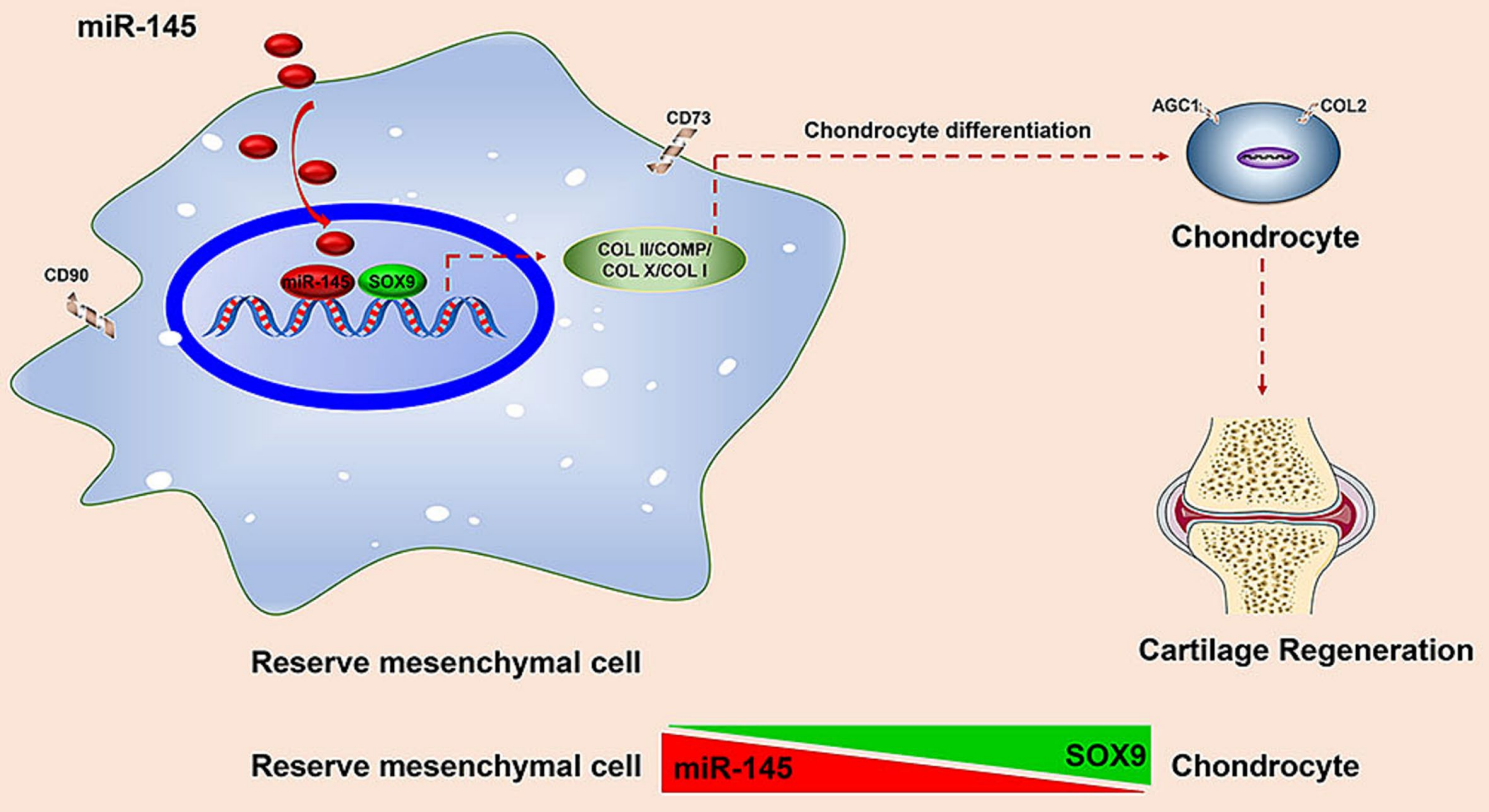
Schematic to illustrate that deer antler reserve mesenchyme cells modified with miR-145 promotes chondrogenesis in cartilage regeneration by targeting SOX9.

This was the first study on the treatment of cartilage defects using miRNA-modified RMCs. We analyzed the expression profile of miRNAs and the biological function of miR-145 during the chondrogenic differentiation of RMCs. We obtained the following results: (1) miR-145 was significantly differentially expressed during the chondrogenic differentiation of RMCs; (2) Knockdown of miR-145 contributed to the phenotype of RMC-derived chondrocytes; (3) miR-145 targets SOX9 to inhibit the phenotype of RMC-derived chondrocytes; (4) RMCs with low expression of miR-145 could differentiate into non-hypertrophic chondrocytes, thereby improving the effectiveness of cartilage repair *in vivo* (Figure 9). Our study provided theoretical support for exploring the mechanism of the regulation of RMCs chondrogenic differentiation by miRNAs, as well as new pathways for the treatment of cartilage lesions by RMC implantation.

Cartilage tissue is mainly composed of proteoglycans and extracellular collagens, with lower cell types and vascular components. Therefore, the healing ability of damaged cartilage is very limited (27). The current methods for treating cartilage defects include pharmacological intervention, surgical intervention, and chondrocyte implantation (28). However, these treatment methods have drawbacks, such as small repair area, fibrocartilage formation, and even cartilage degeneration (29). Recent studies have found that MSC implantation is a promising treatment strategy for promoting cartilage regeneration. Due to the potential for cartilage differentiation, MSCs are very suitable for the treatment of cartilage regeneration (30). Bone marrow mesenchymal cells (BMSCs) are considered the best source of cells for repairing cartilage defects (30). However, the self-renewal ability of BMSCs is limited, characterized by a decrease in their proliferation and differentiation abilities as the donor age increases (31). In addition, BMSCs have a limited lifespan and cannot retain their ability to proliferate and differentiate into cartilage for a long time *in vitro* (32). They may even differentiate into carcinoma-associated fibroblasts (6). In these aspects, RMCs have become the most ideal MSC resources due to their unparalleled proliferative potential, directional differentiation into cartilage lineages, and ability to prevent carcinogenesis (8, 22, 33). However, the regulatory mechanism by which RMCs differentiate into cartilage has not been thoroughly elucidated.

There was growing evidence that miRNAs are crucial in maintaining self-renewal and differentiation in MSCs. Manipulating the expression of specific miRNAs could alter the characteristics of MSCs (32). However, few have explored whether miRNAs are involved in RMC-induced formation and differentiation of cartilage. Therefore, we compared and analyzed the expression profiles of miRNAs during the chondrogenic differentiation of RMCs. Our focus was on finding the characteristics miRNAs that could define the phenotype of different chondrocytes. Ultimately, miR-145 was chosen as a candidate. It was well known that miR-145 had played a regulatory role in the phenotype transition of vascular smooth muscle cells, tumor development, and self-renewal of embryonic stem cells (34–37). However, there were very limited reports on the role of miR-145 in cartilage formation, and even some opposite conclusions have been proposed. On the one hand, it was pointed out that miR-145 inhibited the chondrogenic differentiation of MSCs and caused the degradation of extracellular matrix (ECM) in cartilage tissue. Wu et al. reported that miR-145-5p targeted TLR4 to inhibit chondrogenic differentiation of synovium MSCs (38). Martinez et al. reported that miRNA-145 negatively regulated the function of chondrocytes by targeting SOX9 (39). miR-145 regulates MMP13 upregulation by targeting DUSP6, leading to ECM damage (40). Yang et al. found that miR-145 contributed to impaired ECM in OA cartilage by targeting Smad3 (41). On the other hand, it was supposed that miR-145 could reduce OA-induced chondrocyte death. Wang et al. demonstrated that miR-145 can reduce OA-induced chondrocyte apoptosis by targeting BNIP3 and regulating the Notch signaling pathway (42). Ke et al. reported that miR-145 protected chondrocytes by regulating the miR–FRS2–autophagy axis (43). But so far, no studies have been conducted on the role of miR-145 in RMCs. In the present study, the knockdown of miR-145 in RMCs may effectively promote the chondrocyte differentiation of RMCs, while overexpression of miR-145 may promote hypertrophic differentiation after chondrogenic induction in culture. However, we still need to further explore the regulatory network of miR-145 on chondrogenic differentiation of RMCs.

miR-145 has many target genes, including SOX9, ACAN, FOXO1, and RUNX3 (44). Verbus et al. reported that miR-145 had a corresponding action site in the 3’UTR of the SOX9 gene during chondrogenic differentiation in human MSCs, inhibiting its expression (45). In our study, based on target prediction, qRT-PCR, and dual-luciferase reporter assay, we analyzed the targeted regulatory relationship between SOX9 and miR-145. At mRNA and protein levels, overexpression of miR-145 inhibited the expression of SOX9, while knockdown of miR-145 promoted the expression of SOX9. Therefore, we speculate that SOX9 was a targeted gene of miR-145 in chondrogenic differentiation of RMCs. SOX9 was a transcription factor that triggered the determinative switch to chondrocyte differentiation in MSCs (46). SOX9 was very active during the prechondrocytic mesenchymal condensation stage and maintains high levels of expression in fully differentiated chondrocytes (46). In the rat cartilage injury model, inhibiting the expression of SOX9 greatly inhibited the chondrogenic differentiation ability of BMSCs. Interference with SOX9 expression *in vivo* led to the obstruction of cartilage regeneration (47). In the rabbit cartilage injury model, after overexpression of SOX9, the cartilage marker protein COL2 began to express on the third day of transfection and reached its peak on the 14th day. The results indicated that SOX9 promoted chondrogenic differentiation of BMSCs (48). Zhang et al. found that overexpression of SOX9 could adjust the ratio of COL2 and COL1 in cartilage injury sites, which was similar to the proportion of normal cartilage (49). The above study suggested that SOX9, as a switch for chondrocyte phenotype, could promote the chondrogenic differentiation of MSCs. In our study, SOX9 overexpressing promoted chondrogenic differentiation of RMCs and inhibited the process of hypertrophy. On the contrary, SOX9 knockdown inhibited the chondrogenic differentiation of RMCs. SOX9 knockdown reversed the promoting effect of miR-145 low expression on chondrogenesis of RMCs, and SOX9 overexpression rescued the inhibitory effect of miR-145 overexpressing on chondrogenesis of RMCs. In summary, SOX9 was necessary for the initiation of chondrogenic differentiation of RMCs, and miR-145 targeted SOX9 to inhibit the chondrogenic differentiation process of RMCs.

In addition, this study successfully promoted the repair of cartilage damage by inhibiting miR-145 expression using lentivirus. Previous studies have reported that modifying MSCs with miRNAs is a new approach to enhance the ability of these cells. Lv et al. reported that BMSCs overexpressed with miR-27b can effectively treat OA rats (50). Therefore, miRNA-modified RMCs formed a potentially effective strategy for treating cartilage defects. This study elucidated the regulation mechanism of chondrogenic differentiation of RMCs by miR-145, while also contributing to the efficient repair of cartilage defects by RMC implantation. There are still some limitations to this study. We only focused on miR-145, which cannot regulate the complex process of cartilage defects alone. We also observed other differentially expressed miRNAs, such as miR-140, miR-21, and miR-199a-3p. In addition, miR-145 had many target genes for chondrogenic differentiation of RMCs, such as MTR and KDM6A. Therefore, future studies should focus on identifying common signaling pathways or transcription factors that regulate the expression of miRNAs cluster networks, ensuring a more comprehensive exploration of the important role of RMCs in chondrogenesis. However, due to the injection of cells into the joint, it was not yet clear how many cells were engrafted into the defect. It was recommended to directly implant the cells into the defect. In future research, we will extend the time for RMCs to induce chondrogenic differentiation *in vitro* and cartilage repair *in vivo* to ensure their viability and stability in future applications. To avoid the immune rejection effect of xenograft RMCs, researchers can use the RMC-derived extracellular vesicles, including exosomes and microvesicles, to treat cartilage defects in the future. This will provide new resources for the clinical application of cell-free therapy.

## Conclusion

miR-145 contributed to the balance between endochondral versus chondral differentiation in RMCs by targeting SOX9. This was a preliminary exploration of the mechanism of chondrogenesis and hypertrophy differentiation of RMCs. It has also helped to develop novel approaches that allow for manipulating the differentiation outcome of RMCs for the treatment of cartilage defect.

## Data Availability

The datasets presented in this study can be found in online repositories. The names of the repository/repositories and accession number(s) can be found at: https://www.ncbi.nlm.nih.gov/geo/, GSE277770.

## Supplementary material

Supplementary File S1 Source data of volcano plot of the differentially expressed miRNAs in the pairwise comparisons.

Supplementary File S2 Source data of venn diagram of differentially expressed miRNAs in the pairwise comparisons.

Supplementary File S3 Source data of clustering heatmap of 74 differentially co-expressed miRNAs.

Supplementary File S4 Source data of clustering heatmap of 12 candidate miRNAs related to antler growth.

Supplementary File S5 Source data of venn diagram of the target genes of miR-145 was predicted by online websites.

Supplementary File S6 Source data of venn diagram of differentially expressed target genes of miR-145 in the pairwise comparisons.

Supplementary File S7 Source data of clustering heatmap of 128 differentially co-expressed target genes of miR-145.

Supplementary File S8 **(A)** Quantitative analysis source data of Alizarin blue staining of miR-145 modified RMCs chondrogenic differentiation; **(B)** Quantitative analysis source data of Alizarin blue staining of miR-145/SOX9 modified RMCs chondrogenic differentiation.

Supplementary File S9 **(A)** Quantitative analysis source data of qRT-PCR of FABP4, COL II, COL I of RMCs multilineage Differentiation; **(B)** Quantitative analysis source data of qRT-PCR of miR-145 of RMCs chondrogenic differentiation; **(C)** Quantitative analysis source data of qRT-PCR of COL II, COMP, COL X of miR-145 modified RMCs chondrogenic differentiation; **(D)** Quantitative analysis source data of qRT-PCR of SOX9 of miR-145 modified RMCs chondrogenic differentiation; **(E)** Quantitative analysis source data of qRT-PCR of COL II, COMP, SOX9, COL X of miR-145/SOX9 modified RMCs chondrogenic differentiation.

Supplementary File S10 **(A)** Quantitative analysis source data of IF of COL II, COMP, COL X of miR-145 modified RMCs chondrogenic differentiation; **(B)** Quantitative analysis source data of IF of COL II, COMP, SOX9, COL X of miR-145/SOX9 modified RMCs chondrogenic differentiation

Supplementary File S11 **(A)** Quantitative analysis source data of WB of COL II, COMP, COL X of miR-145 modified RMCs chondrogenic differentiation; **(B)** Quantitative analysis source data of WB of SOX9 of miR-145 modified RMCs chondrogenic differentiation; **(C)** Quantitative analysis source data of WB of COL II, COMP, SOX9, COL X of miR-145/SOX9 modified RMCs chondrogenic differentiation.

Supplementary File S12 **(A)** Macroscopic ICRS scores of the femur; **(B)** Pineda scoring of the HE staining; **(C)** Wakitani scoring of the Safranin-O-Fast green staining.

Figure S1 After 8 weeks of treatment, the grip strength level of rats was investigated using the grip strength test.

Figure S2 The qRT-PCR detection of miR-145 expression after treatment with miR-145 modified RMCs.
