# Supplementary figures and images for: Deer antler reserve mesenchyme cells modified with miR-145 promote chondrogenesis in cartilage regeneration
Source: Front Vet Sci. 2024 Dec 24;11:1500969. doi: 10.3389/fvets.2024.1500969 (PMC11705092; doi:10.3389/fvets.2024.1500969)

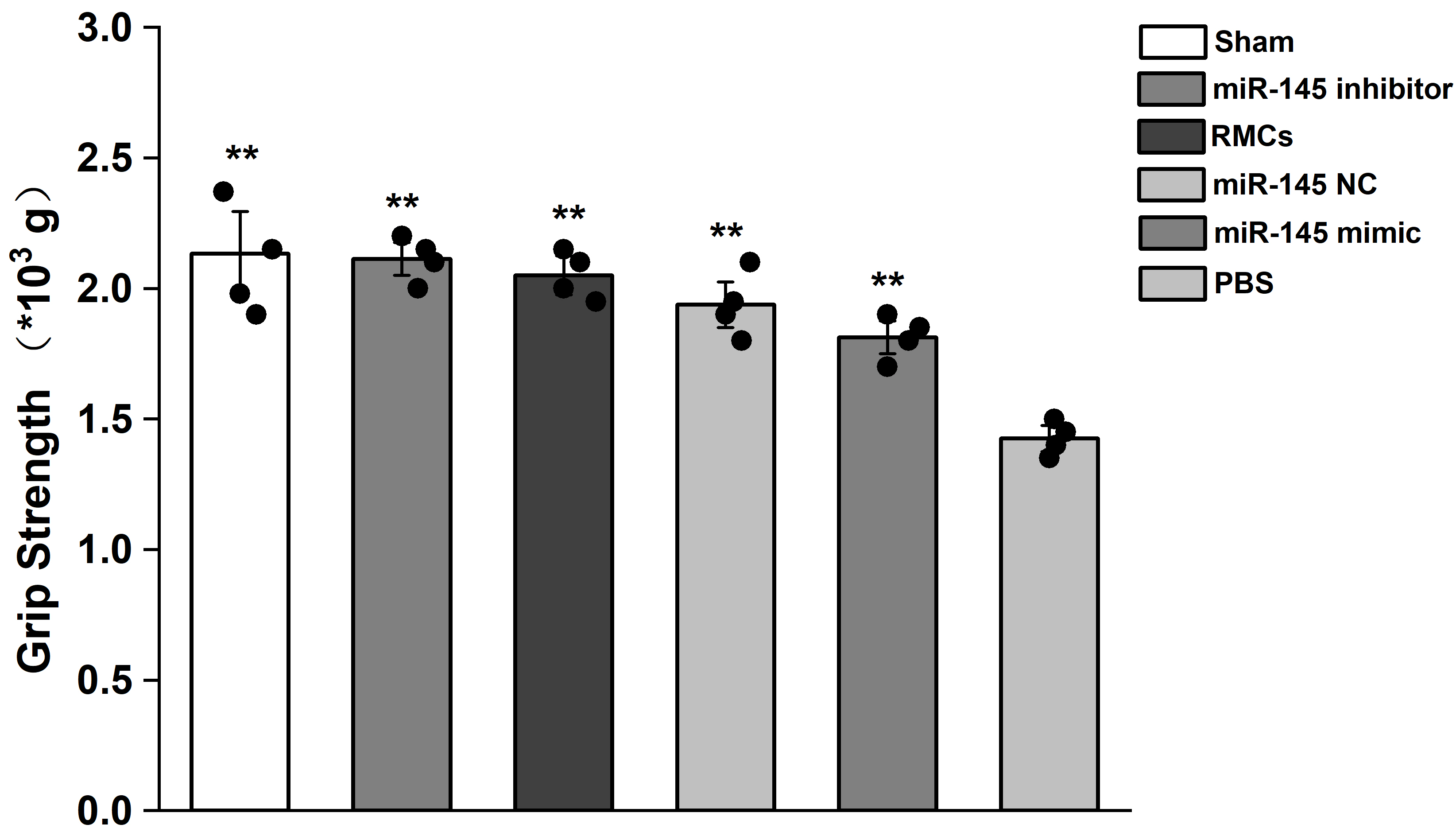

Supplement: Figure S1 — After 8 weeks of treatment, the grip strength level of rats was investigated using the grip strength test. [file Image_1.JPEG]

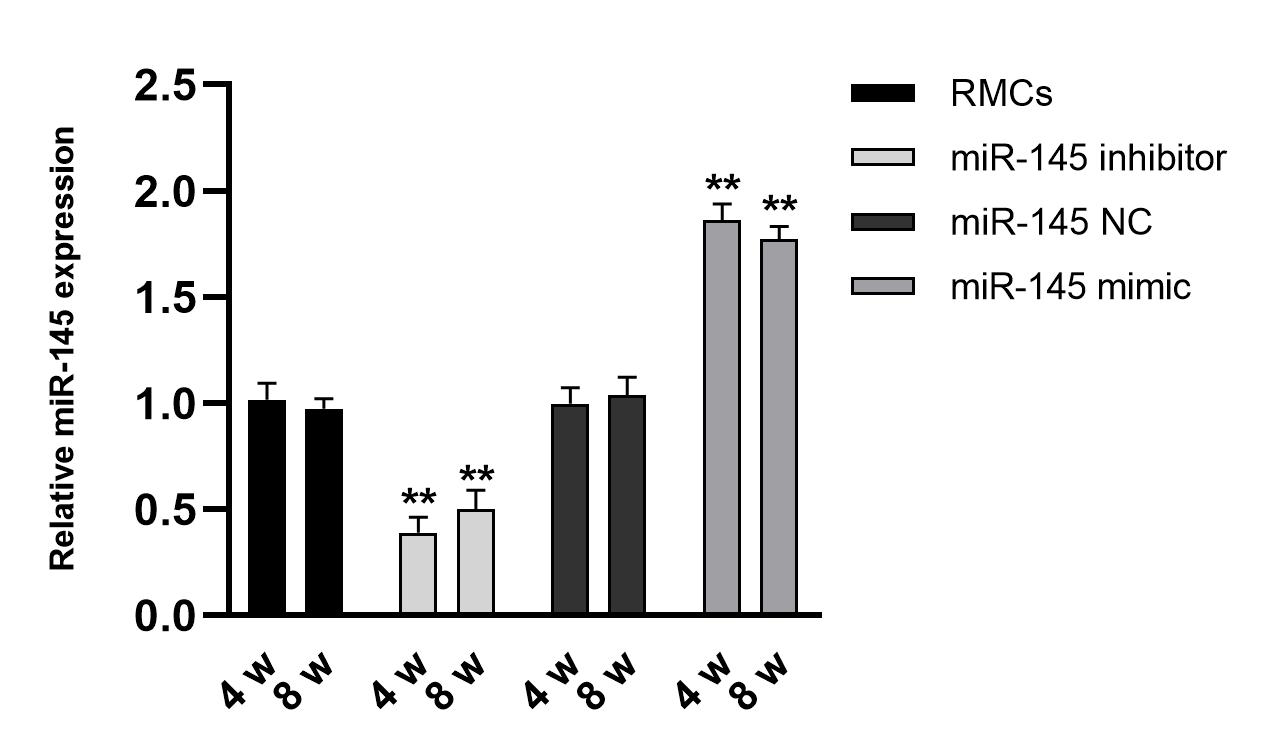

Supplement: Figure S2 — The qRT-PCR detection of miR-145 expression after treatment with miR-145 modified RMCs. [file Image_2.JPEG]
